# Automation in the IVF laboratory in 2026: four technology domains, an asymmetric evidence base, and the case for parallel development with clinician-side automation

**DOI:** 10.1007/s10815-026-03946-7

**Published:** 2026-06-24

**Authors:** Enver Kerem Dirican

**Affiliations:** Department of Obstetrics and Gynecology, Faculty of Medicine, Akdeniz University, Antalya, Turkey

**Keywords:** Artificial intelligence, Embryo selection, Vitrification, Intracytoplasmic sperm injection, Preimplantation genetic testing, End-to-end laboratory automation, Clinician-side automation, Quality management

## Abstract

**Background:**

Operator-dependent laboratory tasks—embryo selection, vitrification and warming, and intracytoplasmic sperm injection (ICSI)—have been the principal targets of IVF laboratory automation, and a fourth strand of work, integrated end-to-end laboratory automation, has produced its first peer-reviewed clinical evidence in 2026. Clinician-side automation in the same ART cycle has an older, broader literature that provides a useful comparator for what laboratory-side automation has and has not yet achieved.

**Objectives:**

To synthesize the published evidence on automation of these laboratory-side task domains; to distinguish what has been demonstrated in randomized or large multicenter studies from what remains in the proof-of-concept phase; to compare the maturity of laboratory-side automation with the older, broader literature on clinician-side automation; to articulate the structural condition—parallel development of clinician-side and laboratory-side automation—that the available evidence suggests would have to be met before any of these technologies could deliver the system-level outcome gains they promise; and to identify a complementary observation that the laboratory tasks for which automation has been most actively developed are not those in which operator competence translates most directly into clinical outcome, with preimplantation genetic testing (PGT) biopsy and tubing as the conspicuous omission.

**Methods:**

A narrative review of PubMed-indexed primary studies, ESHRE/Alpha consensus documents, and Cochrane reviews was conducted, with emphasis on randomized comparisons (Hajek 2021 for vitrification, Illingworth 2024 for AI selection) and large registry or multicenter datasets where available. The 2026 Human Reproduction proof-of-concept report on integrated end-to-end laboratory automation (Chavez-Badiola and colleagues) is treated as the current state of evidence for that domain.

**Key findings:**

(i) Deep-learning embryo grading reproducibly outperforms individual embryologists in retrospective benchmarks across multiple algorithms and centers, but the only published randomized trial failed to demonstrate non-inferiority of deep-learning selection over standard morphology in clinical pregnancy, and the Cochrane review of time-lapse imaging found no evidence of differences in live birth or clinical pregnancy. The realistic value proposition is workflow standardization rather than improved clinical outcome. (ii) Closed semi-automated vitrification (Gavi) achieves clinical and survival outcomes equivalent to manual Cryotop in a multicenter RCT; one-step warming protocols offer substantial workflow gains without compromising survival. (iii) Automated and remotely-operated ICSI have produced healthy live births in proof-of-concept clinical work but remain very early in their clinical adoption curve. (iv) Integrated end-to-end laboratory automation has produced its first proof-of-concept clinical evidence in a single sponsor-conducted pilot in 11 selected patients, in which automated arms were numerically outperformed by manual sibling-oocyte controls; randomized comparison and independent replication are not yet available. (v) Across all four domains, the clinical endpoint of an ART cycle is jointly determined by laboratory-side and clinician-side performance; this co-dependence, examined in detail in the “The clinician’s perspective: where automation has earned trust and where it has not yet” section, places a structural constraint on the system-level benefit any single laboratory-side technology can be expected to deliver. A within-laboratory parallel of this asymmetry is also observed: automation effort has converged on the technically tractable laboratory tasks (ICSI, vitrification, and embryo selection) rather than on the tasks with the highest operator-competence elasticity (PGT biopsy and tubing).

**Conclusion:**

Across the four laboratory-side task domains, the level of clinical evidence available in 2026 is uneven and the maturity gradient is asymmetric with respect to clinician-side automation, which has a longer accumulated evidence base in the same ART cycle. Rather than disappearing, the embryologist’s role is shifting toward verification, exception-handling, and quality oversight. The substantive observation a focused review can make in 2026 is that the system-level benefits projected for laboratory-side automation appear bounded by the slower pace of clinician-side automation development and that the asymmetry between the two halves of the cycle, together with a parallel asymmetry inside the laboratory itself, conditions what any laboratory-side technology can be expected to deliver at the level of patient outcome.

## Introduction

The clinical IVF laboratory is built around operator-dependent tasks that account for the bulk of an embryologist’s day: deciding which embryo from a cohort has the best chance of producing a live birth, cryopreserving and warming oocytes and embryos without compromising developmental potential, and performing ICSI accurately across hundreds of cycles per year. Each is known to suffer from inter- and intra-operator variability [1, 2], and a parallel literature documents comparable inter-clinician variability in the clinician-controlled half of the same ART cycle. The international embryology community has invested considerable effort in standardizing laboratory-side assessment [3, 4], and embryologist workload, fatigue, and burnout have been documented in international surveys [5, 6].

In response, the field has accumulated a substantial corpus of work on automating each task. Deep-learning systems trained on time-lapse images and static blastocyst photographs now match or exceed the performance of individual embryologists in retrospective benchmarks [7–15]. Semi-automated, closed vitrification platforms have been validated against manual Cryotop in a randomized multicenter trial [16], and oocyte vitrification has matured into clinical infrastructure delivering predictable cumulative live-birth rates in fertility-preservation programs [17]. Robotic and digitally controlled ICSI systems have produced their first healthy live births [18, 19]. These technologies have matured at different paces, and the level of supporting clinical evidence remains uneven. A distinct fourth strand integrates these task automations into a single end-to-end automated laboratory; the first peer-reviewed clinical evidence appeared in early 2026 [20] and is treated in the “End-to-end laboratory automation: the Conceivable platform” section. A fifth, integrative question—what these technologies mean for the practicing clinician—is taken up in the “The clinician’s perspective: where automation has earned trust and where it has not yet” section.

This focused review takes a task-centered view, treating AI-assisted embryo selection, semi-automated vitrification, robotic or remote ICSI, and integrated end-to-end laboratory automation as four related but distinct domains. For each, we describe the technology, summarize the strongest available evidence, identify the principal pitfalls, and outline the embryologist’s practical role as the operator–machine boundary moves. We then position these laboratory-side developments alongside the older and more mature literature on clinician-side automation in the same ART cycle and articulate a structural argument—the parallel-development principle—about the relationship between the two.

## AI-assisted embryo selection

### The variability problem that motivates AI

Manual morphological grading remains the most widely used method for choosing which embryo to transfer. The 2011 Istanbul Consensus established common criteria for oocyte, zygote, and embryo assessment and was adopted as the global reference framework for clinical embryology [3]; the 2025 update revisited those criteria with more than a decade of new evidence, including time-lapse data, and provided 20 updated recommendations on preimplantation events and morphological assessment [4].

Despite these efforts, inter- and intra-observer reliability remains bounded. In a multicenter study of ten experienced embryologists choosing a single Day 5 embryo from each of 100 cases, all ten agreed in only half of the cases [1]. A larger multicenter study using the Istanbul Consensus criteria found that inter-center reliability was limited, and that focused training only partially improved agreement [2]. These data establish a baseline against which any automated alternative would need to be evaluated.

### Deep-learning algorithms in retrospective benchmarks

Multiple deep-learning systems have been developed for embryo image and time-lapse video assessment. The Life Whisperer/Genea family of convolutional networks demonstrated higher accuracy than individual embryologists in identifying embryos likely to produce a fetal-heart pregnancy from static blastocyst images [7]. The STORK system, trained on roughly 50,000 time-lapse images, reached an area-under-the-curve above 0.98 for blastocyst quality classification and generalized across centers [8]. Tran and colleagues’ IVY model, trained on more than 10,000 time-lapse video sequences from eight clinics across four countries, predicted fetal-heart pregnancy directly from raw video, illustrating that deep networks can extract predictive features embryologists do not consciously record [9] (Table 1).

**Table 1:** Principal retrospective benchmarks of deep-learning embryo assessment systems

Study	Architecture/modality	Dataset	Outcome assessed	Key finding
VerMilyea et al. [7]	CNN/static blastocyst image	Multi-center	Fetal heart pregnancy	Higher accuracy than individual embryologists in identifying viable blastocysts
Khosravi et al. [8]	Inception CNN/time-lapse images	~ 50,000 images	Blastocyst quality classification	AUC > 0.98; generalized across centers
Tran et al. [9]	CNN/raw time-lapse video	> 10,000 videos, 8 clinics, 4 countries	Fetal heart pregnancy	Predicted directly from video without manual annotation
Chavez-Badiola et al. [10]	CNN/static blastocyst image	Single-center, validated externally	Ploidy and implantation	Accuracy 0.70; PPV 0.79 for euploidy; outperformed senior embryologists in ranking
Loewke et al. [11]	AI ranking/static blastocyst image	Multi-site US clinics	Clinical pregnancy rate	5–12% improvement in CPR vs manual ranking; sensitive to imaging hardware quality
Bormann et al. [12]	Xception CNN + genetic algorithm/time-lapse	3,469 videos (MGH/BWH)	High-quality blastocyst at 113 h.p.i	90% accuracy in identifying high-quality blastocysts
Berntsen et al. [13]	iDAScore v1.0/time-lapse video	Multi-center, externally validated	Embryo ranking for transfer	Performance comparable to manual KIDScore
Theilgaard Lassen et al. [14]	iDAScore v2.0/time-lapse video	181,428 embryos, 22 clinics worldwide	Day 2, 3, and 5 + transfer outcome	AUC 0.62–0.71 by day; performance correlated with morphokinetic parameters
Bori et al. [15]	Automated embryo score/time-lapse	1952 single blastocyst transfers	Implantation and live birth	Score associated with outcome in non-PGT-A cycles; predictive contribution diminished post-PGT-A
Diakiw et al. [21]	CNN/static Day-5 blastocyst image	Multi-center, externally validated	Embryo euploidy (non-invasive)	Predicted euploidy from images; positioned as adjunct, not replacement, for PGT-A

*AUC* area under the receiver operating characteristic curve, *CPR* clinical pregnancy rate, *LBR* live birth rate, *PGT-A* preimplantation genetic testing for aneuploidy

Several centers have developed AI tools operating on static blastocyst images alone, without time-lapse incubation. The ERICA system predicts embryo ploidy and implantation from single static images, achieving 0.70 accuracy with a 0.79 positive predictive value for euploidy and outperforming senior embryologists in side-by-side ranking [10]. Loewke et al. characterized an AI ranking model across multiple US clinics, reporting a 5–12% improvement in clinical pregnancy rate over manual blastocyst ranking in selected sites [11]. Bormann and colleagues developed an Xception-based network with a genetic algorithm and achieved 90% accuracy in identifying high-quality blastocysts at 113 h post-insemination across 3469 time-lapse videos [12].

The iDAScore family, developed and externally validated by Vitrolife, has progressed through two major versions whose performance correlated strongly with morphokinetic parameters, suggesting that the model learned recognizable developmental features rather than spurious clinic-specific patterns [13, 14]. Bori et al., in a single-center series of 1952 single blastocyst transfers, showed that an automatic embryo score derived from time-lapse data was associated with implantation and live birth in cycles without PGT-A, although the predictive contribution diminished once aneuploid embryos had been excluded [15].

A separate strand of work has examined whether deep learning can predict embryo euploidy directly from images. Diakiw et al. developed and externally validated a Day 5 model predicting euploidy likelihood from static images across multiple imaging systems and clinics, intended as a non-invasive ranking adjunct in PGT-A workflows [21]. AI-powered annotation can also standardize interpretation of slow-developing embryos: Cimadomo et al. harmonized developmental-timing and morphological assessment across 1966 Day-7 blastocysts and confirmed that, while less competent than faster-growing embryos, they retain meaningful clinical value for poor-prognosis patients [22].

The technological context for these algorithms is the time-lapse incubator. The 2020 ESHRE Working Group good-practice document explicitly noted that a clear clinical benefit of time-lapse, in terms of increased IVF success, remained to be proven [23]. The most recent Cochrane review of nine RCTs with 2955 participants reached the same conclusion: no evidence of differences in live birth, clinical pregnancy or other reproductive outcomes between time-lapse and conventional incubation, with low to very-low quality evidence [24].

A 2023 systematic review of AI-versus-embryologist comparisons reported that AI systems generally matched or modestly exceeded embryologist performance in retrospective benchmarks but cautioned that almost all studies were retrospective and used different reference standards [25]. Registry data on AI-supported cycles are now emerging [26], and dedicated journal collections have begun to consolidate the methodological landscape [27].

### The Illingworth RCT: a sobering result

The strongest evidence on the clinical value of deep-learning embryo selection comes from a single randomized, double-blind, multicenter non-inferiority trial. Illingworth and colleagues randomized 1,066 women across 14 IVF clinics in Australia and Europe to selection by either iDAScore or standard morphology, with a 5% non-inferiority margin on clinical pregnancy [28]. Clinical pregnancy was 46.5% in the AI arm versus 48.2% in the morphology arm (risk difference − 1.7%, 95% CI − 7.7 to 4.3, *P* = 0.62)—non-inferiority was not demonstrated, although the AI arm was not inferior either. Selection time fell from approximately 208 s per embryo with manual assessment to roughly 21 s with AI, a workflow benefit that may justify adoption even when the clinical pregnancy gain is null. A structural feature further bounds where AI ranking could outperform manual selection: in sequential single-embryo transfers from a frozen cohort, ranking changes the order of transfers but not the set of embryos available, and the patient ultimately receives every viable embryo—so re-ordering cannot in principle change cumulative live birth. The endpoints on which superiority could plausibly be demonstrated are therefore narrower than retrospective benchmarks suggest: time-to-pregnancy, miscarriage rate per transfer, and number of transfers per live birth.

### What AI does and does not change for the embryologist

Several practical conclusions follow. Deep-learning algorithms can rank embryos at least as reproducibly as experienced embryologists, and substantially faster. For clinics with high inter- or intra-observer variability [1, 2], an AI tool may function principally as a standardizing layer rather than as a competence amplifier. In the absence of a positive RCT, clinical adoption should be framed honestly as a workflow tool rather than a fertility-improving device. The embryologist’s role shifts from primary grader to verifier of algorithm output and arbiter of edge cases—borderline blastocysts, atypical morphokinetics, and cases in which clinical context overrides image-based ranking.

## Semi-automated vitrification of oocytes and embryos

### The manual baseline: Cryotop and its operator dependence

Cryotop vitrification, introduced by Kuwayama et al., established the modern minimum-volume cryopreservation paradigm with reported survival above 90% for human oocytes [29]. Two decades on, manual Cryotop remains the dominant clinical method, but the technique requires skilled hand-loading at very small volumes and tight equilibration timing—steps sensitive to operator skill. Open-versus-closed comparisons have been reviewed in detail, with closed systems offering theoretical biosafety advantages at some cost in cooling rate [30]. The practical question has shifted from “which freezing method?” to how to standardize the vitrification step across operators and centers [31].

Inter-operator variability in vitrification outcomes is documented. Gatimel and colleagues compared semi-automated and manual embryo vitrification within a single center and reported reduced inter-operator variability and time savings with the semi-automated approach, with comparable clinical outcomes [32].

### Closed semi-automated vitrification: Gavi

The Gavi platform was developed as the first closed, semi-automated vitrification system aimed at standardizing cryopreservation across operators and centers. Its initial development study reported in vitro outcomes equivalent to manual Cryotop [33]. The pivotal clinical evidence came from Hajek et al., who conducted a randomized, multicenter, open trial comparing Gavi with manual vitrification in women undergoing IVF, with 2PN survival as the primary outcome [16]. Survival was 96.7% with Cryotop and 94% with Gavi; clinical and embryological outcomes were comparable. Procedure time was approximately 47 min for Cryotop versus 81 min for Gavi, reflecting the still-early state of closed-system workflow optimization rather than a fundamental limitation.

These results establish closed semi-automated vitrification as a clinically equivalent option, particularly where standardization across operators is a priority—amultilaboratory network, a training environment, or a quality-management context where reduced variance is the explicit goal.

### Warming protocols and workflow

Warming has its own automation story. Liebermann and colleagues compared a one-step rehydration warming protocol with the standard multi-step protocol in 3,439 frozen embryo transfers and reported identical 99.5% blastocyst survival, with a statistically significant increase in ongoing pregnancy rate (60.4% versus 55.4%, *P* = 0.011) and lower miscarriage rate (4.0% versus 7.6%, *P* = 0.0001) in the one-step group [34]. The simplified protocol reduces warming time, hands-on time and time-out-of-incubator—operational gains that compound across a busy laboratory.

### Oocyte vitrification as fertility-preservation infrastructure

Beyond embryo cryopreservation in conventional IVF, oocyte vitrification has matured into robust clinical infrastructure for fertility preservation. In the largest published series, Cobo and colleagues reported on 6362 women undergoing oocyte vitrification across the IVI-RMA network from 2007 to 2018, including 1073 oncological and 5289 elective cases [17]. Cumulative live-birth rates were closely related to age at vitrification and number of oocytes stored, and were generally consistent with the corresponding rates in fresh autologous IVF. The clinical maturity of oocyte vitrification is what makes elective fertility preservation a reasonable counseling option today rather than an experimental procedure.

### Neonatal and longer-term outcomes after vitrified transfer

Safety data on babies born after vitrified embryo transfer continue to accumulate. In a Belgian cohort of 1072 children born after vitrified embryo transfer, Belva et al. reported neonatal health and congenital malformation rates that did not differ meaningfully from matched fresh-transfer comparisons [35]. Such cohort data are not the final word on long-term safety, but they provide the kind of dataset that allows meta-analyses and guidelines to position vitrification as the de facto standard cryopreservation method it has become [31] (Table 2).

**Table 2:** Principal clinical evidence on vitrification and warming in the modern IVF laboratory

Study	Design	*n*	Comparison/topic	Key finding
Kuwayama et al. [29]	Method development	—	Cryotop minimum-volume vitrification	Established the modern paradigm; > 90% oocyte survival
Vajta et al. [30]	Review	—	Open vs closed systems	Closed offers biosafety advantage at some cost in cooling rate
Rienzi et al. [31]	Systematic review and meta-analysis	Multiple studies	Vitrification vs slow-freezing	Vitrification superior across oocytes, cleavage-stage embryos, and blastocysts; basis for WHO guidance
Roy et al. [33]	In vitro head-to-head	—	Gavi vs manual Cryotop (lab)	Equivalent in vitro outcomes
Hajek et al. [16]	Multi-centre RCT (open)	Multi-center	Gavi vs manual Cryotop (clinical)	2PN survival 94% (Gavi) vs 96.7% (Cryotop); clinical and embryological outcomes comparable
Gatimel et al. [32]	Single-centre comparison	—	Semi-automated vs manual vitrification	Reduced inter-operator variability; time savings; comparable clinical outcomes
Liebermann et al. [34]	Retrospective cohort	3,439 FETs	One-step vs multi-step warming	Blastocyst survival 99.5% in both; ongoing pregnancy 60.4% vs 55.4% (*P* = 0.011); miscarriage 4.0% vs 7.6% (*P* = 0.0001)
Belva et al. [35]	Cohort, neonatal outcomes	1,072 children	Vitrified vs fresh transfer	Neonatal health and malformation rates comparable to fresh-transfer comparisons
Cobo et al. [17]	Registry-scale cohort	6,362 women	Oocyte vitrification, fertility preservation	Cumulative LBR closely related to age and oocyte number; broadly consistent with fresh autologous IVF

*CPR* clinical pregnancy rate, *FET* frozen embryo transfer, *LBR* live birth rate

## Robotic and digitally controlled ICSI

### From manual ICSI (1992) to first robotic prototypes (2011)

ICSI as a clinical procedure dates to Palermo et al.’s 1992 report of pregnancies after intracytoplasmic injection of a single spermatozoon [36]. For three decades, it has been performed manually by highly trained embryologists, with all the operator-dependence implied by multiple distinct micromanipulation steps per oocyte performed under microscope at micrometer precision.

The first robotic ICSI was reported by Lu and colleagues in 2011, who described an automated workstation performing visual sperm tracking, sperm immobilization, picoliter aspiration, and oocyte injection on a hamster-oocyte test system [37]. The work demonstrated technical feasibility but did not address human clinical embryos.

### First clinical babies born after automated ICSI

Costa-Borges et al., working with Conceivable Life Sciences engineers, reported the first babies conceived with automated ICSI [19]. The platform executed the injection sequence—spermatozoon immobilization, capture, and oocyte injection—with embryologist supervision and image-based verification. Although the published evidence rests on early proof-of-concept work rather than randomized comparison, the demonstration that healthy live births are achievable from automated ICSI is itself a substantive milestone.

### Remotely operated ICSI: the Mendizabal-Ruiz case report

Mendizabal-Ruiz et al. reported the first live birth from a digitally-controlled, remotely-operated ICSI procedure, in which the entire multistep micromanipulation sequence was executed under remote-operator command across roughly 3700 km [18]. Mean injection time was 9 min 56 s per oocyte. Five donor oocytes were allocated to the automated remote arm and three sibling oocytes to a manual control arm. Fertilization was 80% (4/5) in the automated arm and 100% (3/3) in the manual arm; two usable blastocysts were generated in each group. Transfer of a warmed blastocyst from the automated arm produced a healthy live birth at 38 weeks.

A single case report does not establish a clinical standard, but it demonstrates that the network-and-control problem—latency, command reliability, error recovery—is now tractable for ICSI. The implications for international training, centralized expert support of isolated clinics, and ultimately full automation are substantial.

## End-to-end laboratory automation: the Conceivable platform

Integrating AI selection, automated vitrification and robotic ICSI into a single end-to-end laboratory workflow—gametes enter after retrieval and the warmed blastocyst is delivered for transfer, with the embryologist supervising rather than executing intervening steps—represents a distinct conceptual move and a distinct level of clinical claim. Conceivable Life Sciences, the same group behind the 2025 remote-ICSI live-birth report [18], has framed this as the goal of its AURA platform, an integrated multi-workstation system bringing sequential IVF procedures under unified automated control. The case for whole-laboratory automation is industrial: broader access to fertility care is likely to depend on engineering laboratory work—largely artisanal since 1978—toward more consistent and scalable execution. The case against is the same recurring throughout this review: the published clinical evidence rests on a single proof-of-concept pilot.

The first peer-reviewed clinical evidence on integrated multisystem automation was published by Chavez-Badiola et al. in Human Reproduction in February 2026 [20]. The study deployed three serially connected automated systems—Pearl 1 (sperm preparation), Pearl 2 (cumulus–oocyte complex retrieval and oocyte denudation), and Pearl 3 (sperm selection, laser immobilization, and piezo-ICSI)—in 11 consenting patients (three autologous, eight donor egg cycles) after minimal or mild stimulation at Hope IVF in Guadalajara, México, between April and October 2024. A sibling-oocyte design allocated some retrieved oocytes to the Pearl pipeline and the remainder to manual processing under identical media and culture conditions. The automated arm achieved 64.3% fertilization (45/70) and 42.2% usable blastocyst formation per zygote (19/45) versus 81% (47/58) and 59.6% (28/47) in the manual arm. Twelve single warmed-blastocyst transfers from the automated arm produced five live births, three biochemical pregnancies and one early loss, for a live-birth rate per transfer of 41.7%. The authors interpreted the pilot as a feasibility demonstration rather than a non-inferiority claim.

Three structural features constrain that interpretation. The design is not powered for non-inferiority, and the manual sibling arm numerically outperformed the automated arm on both laboratory endpoints. The patient population is highly selected—predominantly donor eggs, mild stimulation, and explicit exclusion of severe male-factor infertility and prior fertilization failure—so performance in the clinically harder cases that disproportionately reach tertiary centers has not been tested. The pilot was conducted at a single site institutionally affiliated with the platform’s developer, with operators deeply familiar with the system, and with authorship and sponsorship resting with the developer. The pilot therefore establishes that an end-to-end automated Day 0 workflow can produce viable embryos and healthy live births, but does not establish that it matches manual performance in unselected patients or reproducibly across operators and sites.

### What end-to-end automation would need before routine clinical use

Several thresholds, all of which the AI-selection and semi-automated vitrification literatures have either crossed or are explicitly trying to cross, remain ahead for end-to-end automation. A randomized, multicenter, prospective trial comparing integrated automated workflow to standard manual practice on a clinical endpoint—ideally cumulative live birth, with prespecified non-inferiority margins—is not yet available. Independent, non-sponsor replication at unaffiliated sites has not been published. Cohorts unselected for severe male factor, prior fertilization failure, or low oocyte yield have not been reported. A safety follow-up dataset comparable in size to the Belva cohort for vitrified transfers [35] is not yet available for offspring conceived through end-to-end automated workflows. Each of these is a routine expectation for any laboratory technique proposed for general clinical use, and none is yet met.

## The clinician’s perspective: where automation has earned trust and where it has not yet

The four preceding sections have surveyed automation domain by domain on the laboratory side of the ART cycle. The practicing clinician—who initiates stimulation, monitors response, retrieves oocytes, transfers embryos, and bears clinical and legal responsibility for the cycle—works on the other side of that cycle, and reads laboratory automation through a different lens. This section is written from that lens.

### Automation in the clinician-controlled half of the ART cycle: presence and conspicuous absence

It is worth pausing on a rhetorical asymmetry familiar to anyone attending an IVF-automation meeting. The case for laboratory automation is almost invariably built on the documented inter-operator variability, fatigue, and burnout of embryologists, with the implication that automation is the corrective. The clinician-controlled half of the cycle is rarely subjected to the same diagnostic framing, even though inter-clinician variability in stimulation, retrieval and transfer is comparably documented. The asymmetry of the diagnostic gaze reflects where automation is being marketed and to whom, not an asymmetry of underlying variability.

Automation and decision support have been part of the clinician’s daily practice for at least two decades. Three-dimensional ultrasound with automated antral-follicle counting and follicular volumetry standardizes ovarian-reserve assessment [38]; gonadotropin dose-finding algorithms and trigger-timing models inform stimulation; OHSS risk-prediction models flag patients in whom alternative trigger strategies should be considered; SART- and HFEA-derived prognostic nomograms support counseling; ultrasound guidance for embryo transfer has been the standard of care since the early 2000 s [39]. Each tool has accumulated peer-reviewed validation, has been incorporated into society guidance, and operates inside a settled regulatory and medico-legal framework. They share a structural feature: each produces a recommendation, score, or image overlay that the clinician verifies, integrates with clinical context, and converts into a decision. None executes the decision; none removes the clinician from the loop; none is marketed to patients as an autonomous agent.

A second observation reinforces the point. The two most operator-dependent procedures the clinician personally performs—transvaginal ultrasound-guided oocyte retrieval and intrauterine embryo transfer—have not been meaningfully automated. Robotic biopsy systems for prostate, lung and breast lesions have existed for years; transvaginal robotic oocyte retrieval has not been brought to peer-reviewed clinical reporting. A direct robotic embryo-transfer concept has been described in conference settings but has not produced peer-reviewed clinical outcomes. The laboratory has acquired an end-to-end automation prototype and several first-live-birth case reports, while the clinician’s own micromanipulations remain entirely manual. This asymmetry is not principally a question of technical difficulty but of where commercial pressure currently sits. End-to-end laboratory automation is being developed by companies whose business model removes the embryologist from the loop; equivalent pressure to remove the clinician—the purchaser, prescriber, and holder of medico-legal responsibility—has not emerged.

### The asymmetry between clinician-side and laboratory-side automation

These observations expose a structural difference between the two halves of the ART cycle. Clinician-side automation has converged, over two decades, on a stable model: the tool advises, the clinician decides, and the responsibility is unambiguous. Laboratory-side automation, particularly in its more ambitious recent forms, is drifting away from that model. iDAScore ranks embryos for transfer; AURA performs the Day 0 workflow; remotely operated ICSI executes the micromanipulation steps from another continent. The verbs describe an ambition to move from advising to acting. A move of that magnitude on the clinician side would have been required to traverse the full evidence pathway—randomized comparisons, independent replication, regulatory review, medico-legal clarification—before reaching routine use. On the laboratory side, that pathway has been traversed for vitrification [16, 31, 34, 35] but not yet for AI embryo selection [24, 28], robotic ICSI [18, 19], or end-to-end automation [20].

A related observation reinforces this. Clinician-side automation has implicitly accepted, for two decades, that algorithmic systems operate within definable computational limits [40] and cannot substitute for clinical judgment, positioning them accordingly as decision support requiring human verification. Laboratory-side automation is now testing whether that constraint can be relaxed in practice; the present evidence base does not show that it has been.

### The parallel-development principle

A further structural observation, not previously articulated in the laboratory-automation literature, follows from the same asymmetry. The clinical outcome of an ART cycle—clinical pregnancy, ongoing pregnancy, live birth—is jointly determined by clinician-side and laboratory-side performance, and each side imposes a ceiling on what the other can achieve. An embryo selected by the most sophisticated AI cannot rescue a poorly stimulated cycle, and the most precisely executed transfer cannot rescue a poorly cultured embryo. The chain breaks at its weakest link.

If automation is offered as a remedy for operator-dependent variability and a route to scaled, accessible care, the principle that follows is unambiguous: either both halves of the cycle automate together, or neither does. A laboratory automated to the level of an integrated end-to-end platform [20] paired with a clinician-side workflow still relying on manual stimulation, retrieval and transfer leaves system performance bounded by the manual side. The present evidence is consistent with this prediction: the only RCT of deep-learning embryo selection failed non-inferiority [28], and the only end-to-end pilot was numerically outperformed by manual sibling controls [20]—both conducted where the clinician-side workflow remained entirely conventional. Laboratory automation that outpaces clinician-side automation is unlikely to demonstrate the system-level gains its developers project.

A corollary concerns training and accountability. Inadequate embryologist training degrades cycle outcomes through laboratory error; inadequate clinician training degrades them through stimulation, retrieval, or transfer error of equivalent magnitude—a symmetry sometimes underplayed when laboratory variability is the explicit topic and clinician variability the unspoken background. Workforce sustainability guidance, continuing education, and automation-readiness frameworks would benefit from addressing both sides symmetrically. Parallel investment in robotic oocyte retrieval, AI-supported stimulation protocols, and eventually robotic embryo transfer would create the conditions under which laboratory-side automation could plausibly translate its retrospective benchmarks into clinical-pregnancy and live-birth gains.

### Within-laboratory asymmetry: PGT biopsy and tubing

A version of the same asymmetry is observable inside the laboratory. The four task domains discussed in the “AI-assisted embryo selection,” “Semi-automated vitrification of oocytes and embryos,” “Robotic and digitally controlled ICSI,” and “End-to-end laboratory automation: the Conceivable platform” sections are not equivalent in the magnitude with which operator competence translates into the clinical endpoint. ICSI has converged on a tightly standardized protocol benchmarked by ESHRE/Alpha KPIs [41]; vitrification has comparable maturity, with closed semi-automated platforms reaching clinical equivalence [16]. In both, the marginal clinical effect of remaining inter-operator variability is small. Embryo selection by morphology or AI ranking alters the order rather than the set of embryos transferred and has a bounded effect on cumulative live birth. Trophectoderm biopsy and the subsequent tubing of biopsied cells, by contrast, are precisely the tasks in which embryologist competence translates most directly into clinical outcome: a poorly executed biopsy can damage the blastocyst or contaminate the sample; unsuccessful tubing can render an embryo undiagnosable, so a euploid blastocyst may be discarded as no-result, or a returned result may not reflect the embryo’s actual genetic constitution [42]. The conspicuous feature of the present commercial landscape is that ICSI, vitrification, and embryo selection—the tasks with the lowest operator-competence elasticity—have all attracted dedicated platforms, while PGT biopsy and tubing—the tasks with the highest such elasticity—have attracted essentially none.

### The clinician, the patient, and what “not yet” actually means

The clinician integrates laboratory and clinical information for the patient and holds consent. When a laboratory automation tool is part of the cycle, the consent conversation should describe it at the level of clinical evidence specific to the technology: randomized for vitrification, single RCT not meeting non-inferiority for AI embryo selection, case reports for robotic ICSI, single proof-of-concept pilot for end-to-end automation. “An AI selected your best embryo” misrepresents the “AI-assisted embryo selection” section; “a robot performed your ICSI” misrepresents the “Robotic and digitally controlled ICSI” and “End-to-end laboratory automation: the Conceivable platform” sections. Patients are entitled to the distinction between an embryologist-supervised tool with randomized evidence and one operating under proof-of-concept evidence in a sponsor-conducted pilot. Where the laboratory technology is not yet supported by the evidence the clinician routinely demands of clinical-side innovations, the consent language should reflect that asymmetry honestly.

The position this review converges on is not a rejection of laboratory automation. Vitrification has earned routine adoption [16, 31]. Clinician-side automation has earned its place in stimulation, monitoring, transfer guidance, and counseling. AI embryo selection has earned a workflow-standardization role under embryologist verification [28]. The narrower argument is that end-to-end laboratory automation [20] and AI selection systems intended to operate beyond a verification role have not yet completed the evidence pathway that clinician-side automation completed long ago, and that adoption decisions, regulatory framing, and patient communication should reflect this gap.

## Synthesis: four laboratory-side task domains and a clinician-side framework

Table 3 summarizes the comparative status of the four laboratory-side domains, the within-laboratory PGT exception, and clinician-side automation. The maturity gradient runs from the randomized-trial-supported vitrification literature [16, 17, 31, 34, 35] at one end to the single sponsor-conducted pilot for end-to-end automation [20] at the other, with AI embryo selection [24, 28] and robotic ICSI [18, 19] occupying intermediate positions whose evidence bases differ in kind (large retrospective benchmarks for AI; case reports for robotic ICSI) rather than only in volume.

**Table 3:** Comparative status of automation across the four laboratory-side task domains, the within-laboratory PGT exception, and clinician-side automation. Evidence-level descriptors are based on the strongest published evidence available at the time of writing

Domain	Highest published clinical evidence	Operator competence → outcome elasticity	Commercial automation status	Reference
Vitrification (laboratory)	Multicenter RCT (clinical equivalence)	Low (ultra-rapid protocols largely standardize the procedure)	Mature (Gavi closed system)	[16, 17, 31, 34, 35]
AI embryo selection (laboratory)	Single RCT, non-inferiority not met	Low (cumulative-transfer logic bounds the achievable gain)	Multiple commercial products	[24, 28]
Robotic ICSI (laboratory)	Case reports of first live births	Low (procedure already highly standardized after 30 years)	Early commercial/proof-of-concept	[18, 19]
End-to-end automation (laboratory)	Single sponsor-conducted pilot (*n* = 11)	Mixed (depends on which laboratory step is being executed)	One platform (AURA)	[20]
PGT biopsy and tubing (laboratory)	(Manual standard; technique reviews)	High (operator skill directly affects diagnosis accuracy and embryo viability)	Essentially none	[42]
Clinician-side automation (stimulation, monitoring, transfer guidance)	Multiple RCTs and meta-analyses; standard of care	High (stimulation, retrieval, transfer all highly operator-dependent)	Mature; integrated as decision support	[38, 39]

Two structural features of the synthesis deserve emphasis. First, the spread between the vitrification tier and the active-development tier (robotic ICSI; end-to-end automation) is wider than the spread between the active-development tier and the still-broader gap to mature clinician-side automation—a point visible at a glance in Table 3 and consequential for how any one technology should be communicated to clinicians, patients, and regulators. Second, the within-laboratory pattern is itself instructive: of the laboratory tasks examined here, those that have attracted the most concentrated commercial automation effort are not the tasks on which operator competence translates most directly into clinical outcome.

The framing question this synthesis poses is therefore not which technology to adopt next, but how the development of clinician-side and laboratory-side automation should be coordinated so that the gains projected for either side become available at the level of patient outcome.

## The embryologist’s evolving role

The embryologist’s role is changing across all four domains but not disappearing. In AI selection, the embryologist becomes the verifier, exception-handler, and integrator of clinical context the algorithm does not see. In semi-automated vitrification, the locus of value shifts from manual dexterity to quality oversight, deviation management, and cross-platform method comparison. In robotic ICSI, embryologists supervise the workflow, judge fertilization outcomes, and decide which oocytes are fit for injection. In integrated end-to-end automation, the embryologist is the on-site supervisor responsible for setup, troubleshooting, exception management, and the quality oversight that any sponsor-conducted pilot has so far required to reach published outcomes [18–20].

Quality management infrastructure becomes more important in an automated laboratory, not less. The Vienna Consensus established 19 ART laboratory performance indicators with competence and benchmark values, providing the framework against which any automated technology should be evaluated [41]. The Cairo Consensus set out 50 consensus points on facility design, ventilation, and particulate and VOC control—boundary conditions that no automated workstation can override [43]. The 2025 Istanbul Consensus update anchors the morphological criteria AI tools must respect [4], and the ESHRE good-practice document on time-lapse technology supplies the framework in which most current AI tools are embedded [23].

Workforce data underline the importance of this transition. Comparative survey data on embryologist stress, somatization and burnout in UK HFEA-licensed and US ART/IVF clinics document substantial occupational strain [6], and the staffing-level analysis of Alikani and colleagues quantified the workload required to operate a modern ART unit safely [5]. Automation reduces the cognitively repetitive components of embryology work, so it matters not only for clinical efficiency but for the long-term sustainability of the workforce.

## Limitations and outlook

This is a focused narrative review and not a systematic review; selection of studies prioritizes randomized trials, large multicenter datasets, ESHRE/Alpha consensus documents, Cochrane reviews and milestone proof-of-concept reports and necessarily omits much primary methodology work and many smaller cohort studies. The reference base has been verified against PubMed records but does not pretend to comprehensive coverage of an evolving field.

Five open questions stand out for the next several years.

First, will any AI-assisted embryo selection tool ever pass a positive non-inferiority or superiority trial against “best-practice manual morphology,” or will the field settle for workflow benefits as the principal value proposition?

Second, will closed semi-automated vitrification platforms displace open Cryotop in high-throughput laboratories, or will they remain a parallel option chosen for biosafety and standardization reasons in selected workflows?

Third, will robotic and remote ICSI accumulate a sufficient evidence base—including comparative outcomes and safety follow-up—to move from proof-of-concept toward routine clinical use within the next decade?

Fourth, will the development of clinician-side automation—robotic oocyte retrieval, robotic or AI-guided embryo transfer, and AI-supported stimulation and trigger protocols—proceed at a pace commensurate with the rapid maturation of laboratory-side automation, or will the asymmetry between the two halves of the cycle persist and bound the system-level benefit obtainable from either?

Fifth, will commercial automation development extend to PGT biopsy and tubing—the laboratory tasks in which operator competence translates most directly into clinical outcome—or will the present pattern persist, in which automation effort converges on the more technically tractable laboratory tasks rather than the more clinically consequential ones?

These questions remain unsettled, and the unevenness across them is itself the central practical observation for the next phase of development. The trajectory the available evidence is most consistent with is one in which the system-level outcome gains projected for laboratory-side automation will track the pace at which clinician-side automation develops in parallel; the present pattern, in which laboratory-side automation has advanced faster than clinician-side automation, helps explain why retrospective benchmarks on individual tasks have so far translated into modest signals at the level of cycle outcome. Confidence in any single automation technology will accordingly need to develop incrementally, task by task, and in a manner symmetrical to the way clinician-side decision-support tools have earned their place over the past two decades.

## Data Availability

No datasets were generated or analysed during the current study.
